# Continuous infusion versus intermittent dosing of ceftazidime/avibactam in critically ill patients with *Klebsiella pneumoniae* OXA-48 or *Pseudomonas aeruginosa* infections: a single-center randomized open-label trial (ZAVICONT). Rationale and design

**DOI:** 10.3389/fphar.2025.1618987

**Published:** 2025-08-07

**Authors:** Mirna Momčilović, Ivan Šitum, Ante Erceg, Marko Siroglavić, Mila Lovrić, Laura Nižić Nodilo, Anita Hafner, Jasmina Lovrić, Petra Turčić, Dora Fabijanović, Ana Marinić, Vanja Nedeljković, Marijan Pašalić, Luka Perčin, Dubravka Šipuš, Davor Miličić, Daniel Lovrić

**Affiliations:** ^1^ Department of Cardiovascular Diseases, University Hospital Centre Zagreb, Zagreb, Croatia; ^2^ Department of Anesthesiology, Reanimatology, Intensive Medicine and Pain Therapy, University Hospital Centre Zagreb, Zagreb, Croatia; ^3^ Department of Clinical Microbiology, Infection Prevention and Control, University Hospital Centre Zagreb, Zagreb, Croatia; ^4^ Department of Laboratory Diagnostics, University Hospital Centre Zagreb, Zagreb, Croatia; ^5^ University of Zagreb, Faculty of Pharmacy and Biochemistry, Zagreb, Croatia; ^6^ School of Medicine, University of Zagreb, Zagreb, Croatia; ^7^ Department of Pharmaceutical Technology, University of Zagreb, Faculty of Pharmacy and Biochemistry, Zagreb, Croatia; ^8^ Department of Pharmacology, University of Zagreb, Faculty of Pharmacy and Biochemistry, Zagreb, Croatia

**Keywords:** ceftazidime/avibactam, continuous infusion, critically ill, ICU, *Klebsiella pneumoniae* OXA-48, NCT06811727, *Pseudomona aeruginosa*

## Abstract

**Objective:**

Ceftazidime/avibactam (CZA) is an essential treatment option for managing infections caused by multidrug-resistant (MDR) Gram-negative (G−) bacteria, including *Klebsiella pneumoniae* OXA-48 and carbapenem-resistant *Pseudomonas aeruginosa*. Growing evidence indicates that critically ill intensive care unit (ICU) patients often exhibit altered pharmacokinetics (PK) of CZA, which may compromise the achievement of optimal PK/pharmacodynamic (PD) targets with standard dosing regimens. The primary hypothesis of this study is that continuous infusion (CI) of CZA improves microbiological success compared to intermittent dosing (ID) in critically ill ICU patients with severe infections caused by *K. pneumoniae* OXA-48 or *P. aeruginosa*.

**Methods:**

This is a single-center, randomized, open-label trial with a 1:1 allocation ratio, conducted at the University Hospital Centre Zagreb, a tertiary care hospital in Croatia. A total of 140 critically ill ICU patients with severe infections due to *K. pneumoniae* OXA-48 or *P. aeruginosa* requiring CZA treatment will be randomized to receive either ID of CZA (2 g/0.5 g/8 h over 2 h) or the same total daily dose in CI (6 g/1.5 g over 24 h). The study is powered to demonstrate the superiority of CI over ID of CZA in terms of microbiological success.

**Outcomes:**

The primary outcome will be microbiological success rate, chosen as a key indicator of pathogen eradication that is directly influenced by PK/PD target attainment. Secondary outcomes will include clinical success rate, time to symptoms improvement, length of ICU stay, length of hospital stay, all-cause 28-day mortality, pathogen recurrence rate on day 28, time to weaning from mechanical ventilation, cumulative vasoactive-inotropic score, adverse events, and the ratio of ceftazidime plasma concentration to the pathogen’s minimum inhibitory concentration (C/MIC).

**Conclusion:**

This trial will provide evidence on optimal CZA administration regimen in critically ill ICU patients with severe infections due to MDR G-pathogens.

**Clinica Trial Registration:**

clinicaltrials.gov, identifier NCT06811727.

## 1 Introduction

Ceftazidime/avibactam (CZA) is an essential treatment option for managing infections caused by multidrug-resistant (MDR) Gram-negative (G−) bacteria, including complicated intra-abdominal infections (cIAI), complicated urinary tract infections (cUTI), hospital-acquired pneumonia (HAP), ventilator-associated pneumonia (VAP), and associated bacteremia (European Medicines Agency, 2024). Approved by the Food and Drug Administration (FDA) in 2015 and the European Medicines Agency (EMA) in 2016, CZA effectively targets key pathogens like *Enterobacteriaceae* (e.g., *Klebsiella*) and *Pseudomonas aeruginosa* (European Medicines Agency, 2024), which have become a significant global healthcare challenge due to rising carbapenem resistance. Studies have highlighted the genetic diversity of carbapenem-resistant *Klebsiella pneumoniae* (CRKP), with subspecies producing New Delhi metallo-β-lactamase (NDM) and oxacillinase-48 (OXA-48)-like carbapenemases, with varying lineages [e.g., sequence type (ST)-14, ST-16, ST-231] (Ceron et al., 2023; Garcia-Gonzalez et al., 2023; Chen et al., 2023). The Mediterranean region is particularly noted for endemic strains of *Klebsiella* that produce OXA-48 oxacillinase (Bonomo et al., 2018). *P. aeruginosa* resistance to carbapenems is driven by the production of MBLs (VIM, IMP, NDM, SPM), mutations in porin channels and the overexpression of efflux pumps (Poole, 2011).

Recent European data underscore the growing threat. For *K. pneumoniae*, data from the European Antimicrobial Resistance Surveillance Network (EARS-Net) for 2023 shows a significantly increasing trend of population-weighted mean percentage of carbapenem (imipenem or meropenem) resistant isolates from 10.4% in 2019 to 13.3% in 2023 (European Centre for Disease Prevention and Control, 2023a). Significantly increasing national trends in carbapenem resistance percentages were observed in invasive isolates of *K. pneumoniae* during the period 2019–2023 for Croatia and many other European countries (European Centre for Disease Prevention and Control, 2023b). Estimated incidence of bloodstream infections (BSIs) of CRKP for the EU/EEA increased from 2.52 in 2019 to 3.97 per 100,000 population in 2023, which constitutes an increase of 57.5% (European Centre for Disease Prevention and Control, 2023a). Estimated incidence of BSIs of carbapenem-resistant *P. aeruginosa* for the EU/EEA increased from 1.73 in 2019 to 2.01 per 100,000 population in 2023, which constitutes an increase of 16.2% (European Centre for Disease Prevention and Control, 2023a). Carbapenem-resistant *P. aeruginosa* is listed by World Health Organisation (WHO) as a pathogen of high priority that requires research and development of new antibiotics (World Health Organization, 2024).

The rising prevalence of MDR pathogens, combined with limited treatment options and high associated mortality, highlights the urgent need for optimized therapeutic strategies and infection control. Effective antibiotic regimens play a crucial role in combating resistant bacterial strains, whereas antibiotic resistance poses a significant threat to public health (Batchelder et al., 2023). Strategies like antibiotic cycling, evolutionary steering, and combination therapies have been proposed to slow down resistance development and enhance treatment outcomes (Ghazi and El Nekidy, 2023). Additionally, the rediscovery and optimization of dosing of older antibiotics have shown promise in treating infections caused by resistant bacterial strains, albeit with considerations for potential adverse effects (Ali et al., 2022). Avibactam is a potent β-lactamase inhibitor, making CZA active against 94%–100% of *Klebsiella Pneumoniae*-carbapenemase (KPC) and OXA-48-producing pathogens from the *Enterobacteriaceae* family (Tamma et al., 2023; Kazmierczak et al., 2018). However, resistance to CZA has also been observed (Cui et al., 2023), necessitating the exploration of dosing strategies that optimize pharmacokinetic/pharmacodynamic (PK/PD) targets.

Antimicrobial activity of CZA is primarily defined by the percent time of the free-drug concentration above the CZA minimum inhibitory concentration (MIC) for ceftazidime, and the percent time of the free-drug concentration above a threshold concentration (Ct) for avibactam (Das et al., 2019), over the dosing interval. Built on previous findings, including 50% fT > MIC as a well-established target for the efficacy of ceftazidime alone (Muller et al., 2013), MIC values for CZA ranging from 4–8 mg/L for *P.aeruginosa* (Nichols et al., 2016; Flamm et al., 2014) and 0.5–4 mg/L for *Enterobacteriaceae* (Flamm et al., 2014; De Jonge et al., 2016), and threshold concentration for avibactam of at least 1 mg/L to be effective for both *Enterobacteriaceae* and *P.aeruginosa* (Coleman et al., 2014; Berkhout et al., 2016), the joint PK/PD target for CZA was established (50% fT > 8 mg/L for ceftazidime and 50% fT > Ct of 1 mg/L for avibactam) (Das et al., 2019). The selected target was used in population pharmacokinetic modelling to determine a dosing regimen of 2 g/0.5 g every 8 h via a 2-h intravenous infusion, for all the aforementioned indications. The recommended regimen, with dose adjustment in patients with creatinine clearance (ClCr) < 50 mL/min (Das et al., 2019), has been evaluated in five randomised Phase III clinical trials (RECLAIM 1 and 2, RECLAIM 3, REPRISE, RECAPTURE, REPROVE) (Mazuski et al., 2016; Wagenlehner et al., 2016; Carmeli et al., 2016; Qin et al., 2017; Torres et al., 2018), that all confirmed its efficacy and safety. A recent *post hoc* pooled analysis of these trials further reinforced the clinical effectiveness and safety of CZA in patients with infections caused by ESBLs, AmpC and serine carbapenemase-producing G-pathogens (Torres et al., 2023). However, its suitability for critically ill patients in intensive care units (ICUs) with altered PK profiles remains unclear. Early post-marketing evaluations highlighted the urgent need for PK investigations in this high-risk population to optimize dosing, minimize risk of underexposure, and prevent the emergence of resistance (Falcone and Paterson, 2016). Currently approved intermittent dosing (ID) regimens for β-lactams generally have more than an 80% probability of achieving the PK/PD target, but data for ICU patients are very limited. It is believed that a target of 100% fT ≥ 4×MIC, and according to some sources, even 100% fT ≥ 5×MIC in patients with severe infections in the ICU would contribute to clinical efficacy (Al Shaer et al., 2020; Roberts et al., 2014). It is also important to note that the PK/PD target necessary to prevent resistance is often higher than the one needed for clinical efficacy. In critically ill patients, factors such as increased capillary permeability, volume redistribution, large fluid infusions, low serum albumin, and interventions like mechanical ventilation can alter β-lactam pharmacokinetics, notably the volume of distribution (Vd) and clearance (Cl). Renal clearance is often reduced but may also be elevated, especially in sepsis, further complicating drug distribution, particularly in septic shock where microcirculatory impairment reduce drug concentration at infection sites. Hydrophilic drugs like ceftazidime are particularly susceptible to fluctuating plasma levels, making standard dosing regimens, derived from healthy volunteers’ PK data, potentially inadequate for critically ill patients. The effectiveness of β-lactams can be difficult to measure early on, especially within the first 24–48 h of therapy, and therapeutic drug monitoring (TDM) is not widely available. This can obscure whether poor outcomes are due to illness severity or underdosing in ICUs.

Studies reveal significant pharmacokinetic variability in ceftazidime among ICU patients with *P. aeruginosa* infections and severe sepsis (Rondanelli et al., 1986; Young et al., 1997; Gómez et al., 1999). Notably, substantial interindividual differences in half-life (t1/2) and trough levels of ceftazidime reflect variations in Vd and Cl (Rondanelli et al., 1986; Young et al., 1997). Even in pediatric ICU patients, recent population PK data showed high variability in ceftazidime Cl, with body weight and eGFR identified as key covariates (Li et al., 2024). Given that β-lactams’ bactericidal effect depends on the time that drug concentration exceeds MIC, extending infusion duration may counter pharmacokinetic shifts, keeping plasma levels above MIC for a longer time. It has been confirmed that the continuous infusion (CI) of ceftazidime in critically ill patients with HAP maintains clinical efficacy, optimizes the PD profile, ensures concentrations above the MIC for a longer period (Cousson et al., 2005; Nicolau et al., 2001), and allows for the use of a smaller total dose of the drug (Nicolau et al., 2001; Angus et al., 2000) compared to ID. Greater clinical efficacy of ceftazidime in CI compared to ID has been demonstrated in patients with VAP caused by G-pathogens (Lorente et al., 2007). A pharmacokinetic study by El Haj et al. (2024) demonstrated that CI also provided superior anti-biofilm activity *in vitro* compared to ID in both susceptible and resistant *P. aeruginosa* strains, highlighting the PD benefits of sustained drug exposure. A pharmacoeconomic comparison of continuous versus intermittent ceftazidime use showed that CI is a cost-effective alternative to ID (McNabb et al., 2001).

Growing evidence indicates that critically ill patients are also at an increased risk of altered PK of CZA and that optimal PK/PD exposures may not be achieved with standard dosing regimens (Bakdach et al., 2022; Cojutti et al., 2024). Suboptimal exposure to CZA can contribute to the development of resistance in *Enterobacteriaceae* pathogens, particularly in critically ill patients (Adembri et al., 2020). This was demonstrated in a case report of a critically ill patient with KPC-producing *K. pneumoniae* bacteremia where suboptimal CZA exposure led to pathogen mutation and the development of resistance (Gaibani et al., 2021). Gatti and Pea suggested that implementing modified dosing strategies such as extending infusion duration or increasing doses, together with TDM, could be the most effective approach to optimizing PK/PD in critically ill patients (Gatti and Pea, 2021a). This strategy may also aid in eradicating difficult-to-treat (DTR) G-pathogens, reduce the risk of microbiological treatment failure, and prevent unnecessary use of combination therapy (Gatti and Pea, 2021a; Gatti et al., 2023a; Gatti et al., 2023b; Situm et al., 2024). Pharmacokinetic analyses further support that CI can overcome dosing limitations, particularly for pathogens requiring aggressive PK/PD targets (Cojutti et al., 2024). Continuous infusion could also reduce the total daily dose, as lower doses than those recommended in the standard dosing regimens may still achieve the desired PK/PD target (Gatti et al., 2023b; Fresan et al., 2023). A review of real-world data from 2021 found that prolonged infusion (3 h) of CZA was negatively associated with mortality; however, only 23.7% of patients were hospitalized in the ICU (Gatti and Pea, 2021b).

Despite growing evidence supporting CI of CZA in critically ill patients, current studies remain limited by small sample sizes, observational designs, and variability in dosing regimens. Consequently, robust, high-quality evidence is still lacking to definitively establish the clinical superiority of CI over ID in this population. The primary hypothesis of this study is that CI of CZA improves microbiological success compared to ID in critically ill ICU patients with severe infections due to *Klebsiella pneumoniae* OXA-48 or *Pseudomonas aeruginosa*. The central research question is whether CI of CZA improves microbiological outcomes compared to ID in this high-risk population.

## 2 Materials and methods

### 2.1 Study design

ZAVICONT is a single-center, randomized, open-label trial with 1:1 allocation ratio. It is registered on ClinicalTrials.gov with identifier number NCT06811727. The study design was guided by the Population, Intervention, Comparator, Outcome (PICO) framework, outlined in detail in Table 1.

**TABLE 1:** PICO framework used to guide ZAVICONT study design.

P	Population	Critically ill ICU patients with severe infections caused by *Klebsiella pneumoniae* OXA-48 or *Pseudomonas aeruginosa*
I	Intervention	Continuous infusion of ceftazidime/avibactam
C	Comparison	Intermittent dosing of ceftazidime/avibactam (as per SmPC)
O	Outcome (s)	Primary outcome: microbiological success rate Secondary outcomes: clinical success rate, time to symptoms improvement, length of ICU stay, length of hospital stay, all-cause 28-day mortality after ceftazidime/avibactam initiation, pathogen recurrence rate on day 28, time to weaning from mechanical ventilation, cumulative vasoactive-inotropic score, adverse events, ratio of ceftazidime plasma concentration to the pathogen’s minimum inhibitory concentration (C/MIC)

Abbreviations: ICU, intensive care unit; SmPC, summary of product characteristics.

### 2.2 Study aim

The aim of this study is to investigate efficacy of continuous infusion of ceftazidime/avibactam compared to conventional intermittent dosing, in treating critically ill ICU patients with severe infections caused by *Klebsiella pneumoniae* OXA-48 or *Pseudomonas aeruginosa.* The primary outcome of the study is microbiological success rate, defined by proportion of patients in whom the causative pathogen is absent from specimen at the site of infection.

Secondary outcomes are as follows:

a) clinical success rate
b) time to symptoms improvement
c) length of ICU stay
d) length of hospital stay
e) all-cause 28-day mortality after ceftazidime/avibactam initiation
f) pathogen recurrence rate on day 28
g) time to weaning from mechanical ventilation
h) cumulative vasoactive-inotropic score (VIS)
i) adverse events
j) ratio of ceftazidime plasma concentration to the pathogen’s minimum inhibitory concentration (C/MIC)

### 2.3 Population

We plan to enroll 140 critically ill ICU patients with severe infections and with at least one microbiological sample positive for *Klebsiella pneumoniae* OXA-48 or *Pseudomonas aeruginosa*, requiring by clinical judgement antibiotic therapy with ceftazidime/avibactam. Key inclusion and exclusion criteria are listed in Table 2. Participants are eligible to be included only if all inclusion criteria are met. Participants are excluded from the study if any of the listed exclusion criteria apply. The informed consent is obtained from the patient if the patient is capable of making a decision about whether to accept the participation in the study. In cases where the patient has altered consciousness or is unconscious, the decision regarding participation is made by the patient’s next of kin. If an alternative decision maker is unavailable, the decision to enroll the patient can be made by consensus of three or more intensive care physicians, as per the request of the Ethics Committee of UHC Zagreb. The consent to continue the study will be collected when the clinical conditions permit or when an alternative decision maker becomes available.

**TABLE 2:** ZAVICONT key inclusion and exclusion criteria.

Inclusion criteria
General
1. Age above or equal to 18 years
2. Able to provide informed consent personally or by him/her next of kin, as requested by Ethics Committee
Disease specific
3. Critically ill patients requiring admission to intensive care unit (medical or surgical)
4. Diagnosed with severe infections
5. At least one microbiological sample positive for *Klebsiella pneumoniae* OXA-48 or *Pseudomonas aeruginosa*
6. Requiring a prescription for ceftazidime/avibactam, by clinical judgement
Exclusion criteria
General
1. Known or suspected hypersensitivity to ceftazidime/avibactam, any of the excipients or any other cephalosporin antibacterial agent. Severe hypersensitivity (e.g., anaphylactic reaction, severe skin reaction) to any other β-lactam antibacterial agent (e.g., penicillins, monobactams or carbapenems)
2. Withdrawal of informed consent
3. Age above 85 years of age
4. Female who is pregnant or breast-feeding
5. Participation (i.e., signed informed consent) in any other interventional clinical trial of an approved or non-approved antibacterial agent within 30 days prior to screening
6. Any disorder, which in the investigator’s opinion might jeopardize participant’s safety or compliance with the protocol
7. Primary pathogen *Pseudomonas aeruginosa* susceptible to meropenem or imipenem
Laboratory values
8. Severe neutropenia prior or during ceftazidime/avibactam administration
Medical conditions
9. Death within 48 h following randomization
10. Concomitant acquired immunodeficiency syndrome
11. Presence or history of malignant neoplasms or *in situ* carcinomas
12. Duration of ceftazidime/avibactam administration is shorter than 72 h

### 2.4 Randomization and allocation

In this research, we will use block randomization in 1:1 ratio, with a block size of six to ensure an even distribution of subjects between the control and intervention group. The randomization list was generated in advance using a computer algorithm with a fixed seed for reproducibility of results. Block randomization was chosen in order to achieve a balance in the size of the groups throughout the duration of the study, which is especially important due to the planned interim analysis after the inclusion of the first 34 subjects per group. The final goal is to include a total of 70 patients per group. Randomization will be performed by the ICU physician in charge of the patient, after confirming the patient’s eligibility. To maintain study integrity, patients will not be informed of their group assignment. We opted for this study design, which is not fully blinded, for several practical and clinical reasons. First, the dosing regimen of the drug is complex. According to the SmPC, ceftazidime/avibactam is administered as a prolonged infusion over 2 h every 8 h. In a placebo-controlled design, all patients would require an additional infusion, either placebo or the active drug, following the initial 2-h infusion. By not including a placebo, the control group will follow the SmPC dosing regimen (2-h infusions every 8 h), while the intervention group will receive the drug as a continuous infusion over 24 h. Second, the study involves administration in cardiac and cardiac surgery intensive care units, where patients are at bigger risk of volume overload. To minimize unnecessary fluid administration, the study was designed without a placebo-controlled arm. Finally, under local regulations, antibiotics are prepared and administered by nurses. Due to limited nursing staff and shift rotations, which involve multiple staff members in the care of a single patient, using a placebo would significantly increase the workload. To address this logistical challenge and ensure feasibility, placebo was excluded from the study design. In order to reduce biases, data collection will be performed by trained personnel who will not participate in patient care and will be blinded to group allocation. In addition, outcome assessors will be blinded to treatment assignment to ensure objective evaluation of clinical and microbiological endpoints.

### 2.5 Interventions

**FIGURE 1:**
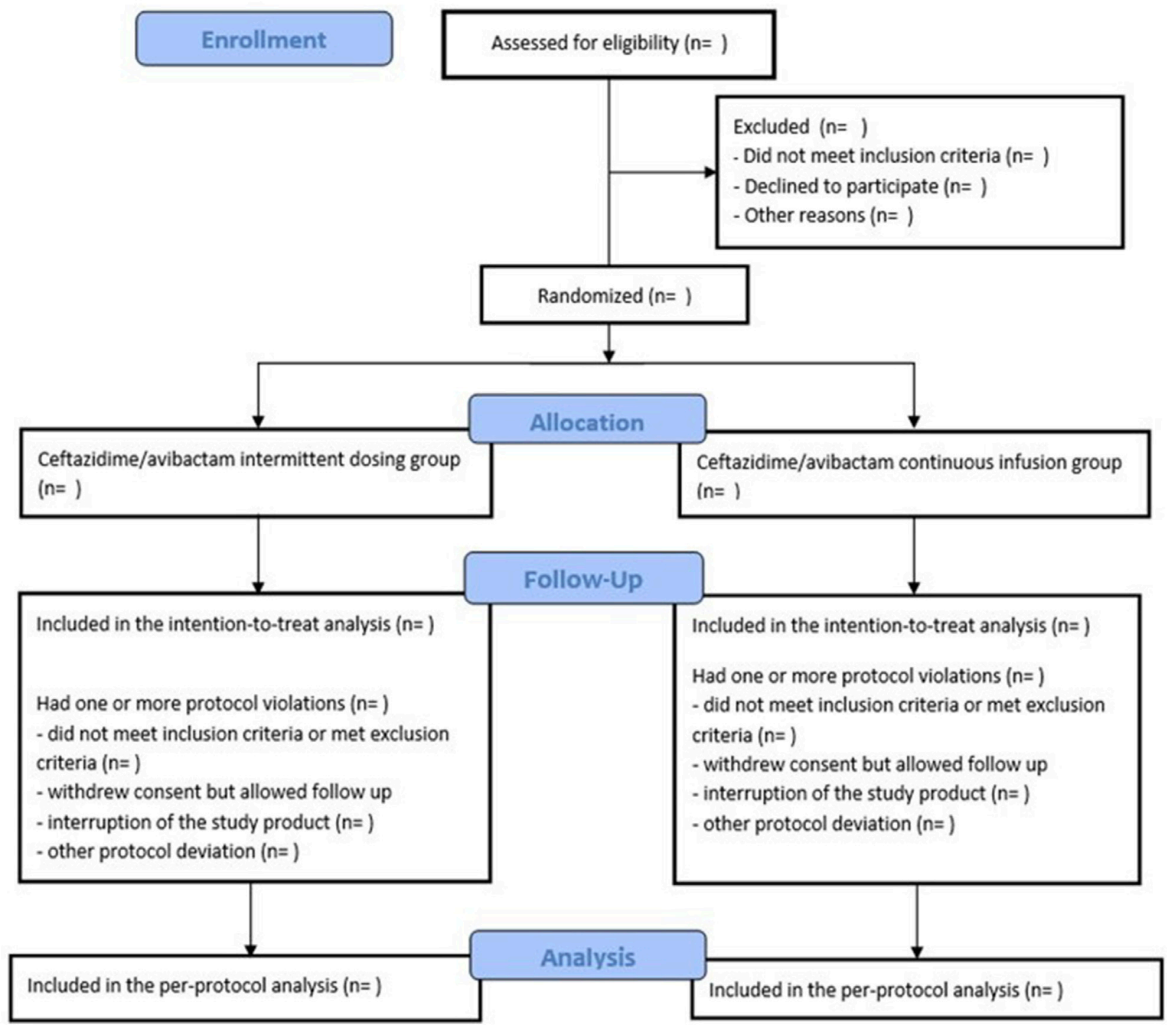
ZAVICONT trial flowchart.

**FIGURE 2:**
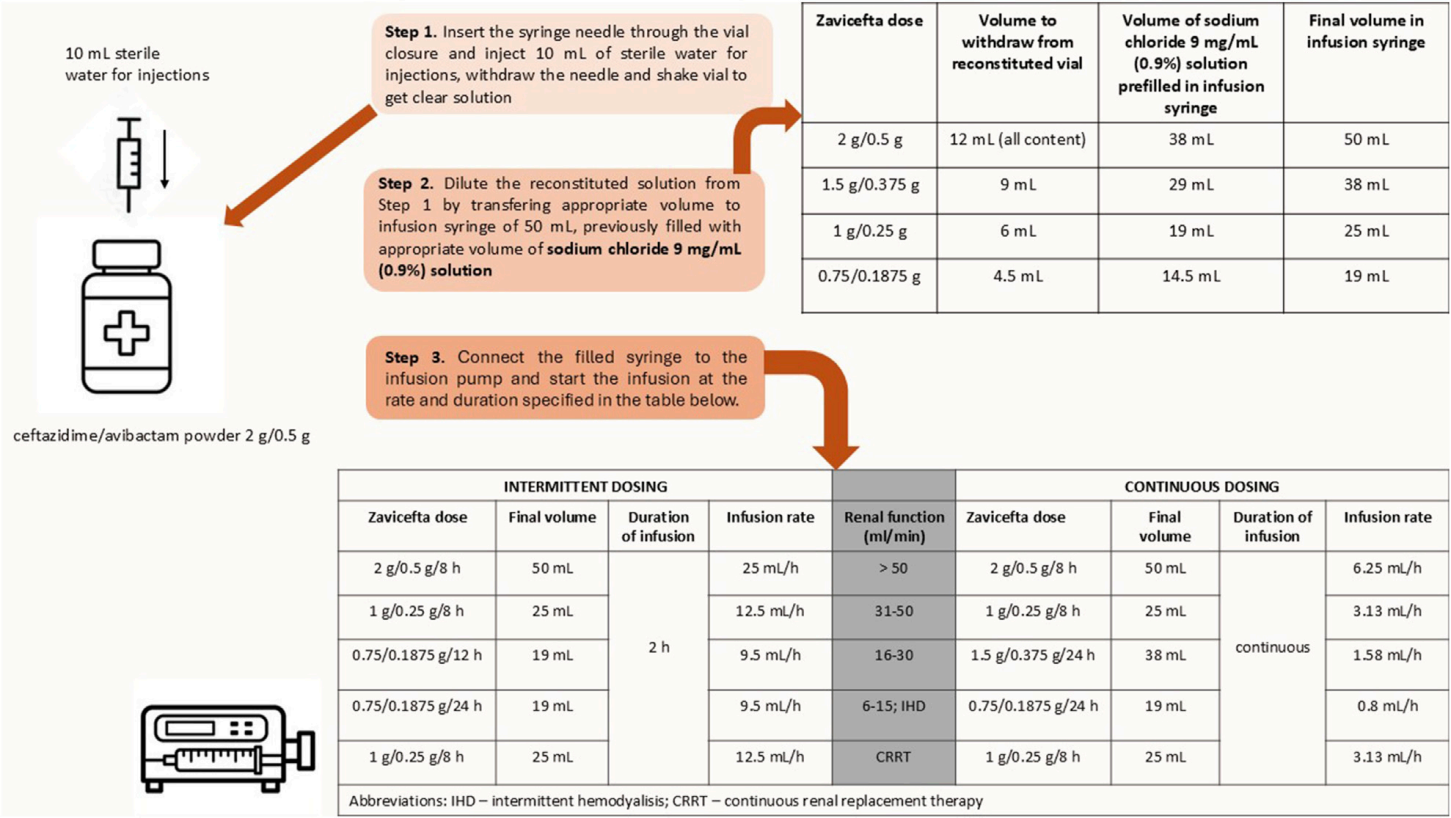
Drug reconstitution, dilution and infusion settings.

Participants will be randomized to receive either intermittent dosing or continuous infusion of ceftazidime/avibactam (Figure 1). Intermittent dosing, as outlined in the SmPC, consists of 2 g/0.5 g administered by prolonged infusion over 2 h every 8 h (European Medicines Agency, 2024). Continuous infusion will include a loading dose of 2 g/0.5 g administered over 2 h, followed by continuous infusion of 6 g/1.5 g over 24 h, equivalent to 0.25 g of ceftazidime per hour. The drug reconstitution and dilution process are shown in Figure 2. The final volume of solution of CZA will be 50 mL, which gives concentration of ceftazidime of 40 mg/mL, with 4:1 concentration ratio for avibactam (10 mg/mL). The solution will be administered via an infusion syringe, with an infusion rate of 6.25 mL/h. Dose adjustments will be applied according to renal function, calculated using Cockroft-Gault formula.

Therefore, patients in control group will receive study drug according to schedule:

a) for creatinine clearance >50 mL/min: 2 g/0.5 g in 50 mL sodium chloride 9 mg/mL (0.9%) solution for injection every 8 h administered over 2 h at infusion rate 25 mL/h (total daily dose 6 g/1.5 g)
b) for creatinine clearance 31–50 mL/min: 1 g/0.25 g in 25 mL sodium chloride 9 mg/mL (0.9%) solution for injection every 8 h administered over 2 h at infusion rate 12.5 mL/h (total daily dose 3 g/0.75 g)
c) for creatinine clearance 16–30 mL/min: 0.75 g/0.1875 g in 19 mL sodium chloride 9 mg/mL (0.9%) solution for injection every 12 h administered over 2 h at infusion rate of 9.5 mL/h (total daily dose 1.5 g/0.375 g)
d) for creatinine clearance 6–15 mL/min and intermittent haemodialysis (thrice weekly): 0.75 g/0.1875 g in 19 mL sodium chloride 9 mg/mL (0.9%) solution for injection every 24 h administered over 2 h at infusion rate 9.5 mL/h* (*administered after completion of haemodialysis) (total daily dose 0.75 g/0.1875 g)
e) for continuous renal replacement therapy (CRRT): 1 g/0.25 g in 25 mL sodium chloride 9 mg/mL (0.9%) solution for injection every 8 h administered over 2 h at infusion rate 12.5 mL/h (total daily dose 3 g/0.75 g)

Patients in intervention group will receive study drug according to schedule:

a) for creatinine clearance >50 mL/min: 2 g/0.5 g in 50 mL sodium chloride 9 mg/mL (0.9%) solution for injection every 8 h administered in CI over 8 h at infusion rate 6.25 mL/h (total daily dose 6 g/1.5 g)
b) for creatinine clearance 31–50 mL/min: 1 g/0.25 g in 25 mL sodium chloride 9 mg/mL (0.9%) solution for injection every 8 h administered in CI over 8 h at infusion rate 3.13 mL/h (total daily dose 3 g/0.75 g)
c) for creatinine clearance 16–30 mL/min: 1.5 g/0.375 g in 38 mL sodium chloride 9 mg/mL (0.9%) solution for injection every 24 h administered in CI over 24 h at infusion rate 1.58 mL/min (total daily dose 1.5 g/0.375 g)
d) for creatinine clearance 6–15 mL/min and intermittent haemodialysis (thrice weekly): 0.75 g/0.1875 g in 19 mL sodium chloride 9 mg/mL (0.9%) solution for injection every 24 h administered in CI over 24 h at infusion rate 0.8 mL/h (total daily dose 0.75 g/0.1875 g)
e) for continuous renal replacement therapy (CRRT): 1 g/0.25 g in 25 mL sodium chloride 9 mg/mL (0.9%) solution for injection every 8 h administered in CI over 8 h at infusion rate 3.13 mL/h (total daily dose 3 g/0.75 g)

In several sources, the short-term stability of CZA in aqueous solution after reconstitution and dilution is noted as a potential issue for continuous administration. CZA is stable in infusion syringes for up to 6 h at temperatures not exceeding 25°C within concentration range 8–40 mg/mL (European Medicines Agency, 2024). This is insufficient for administration via continuous infusion lasting 8–24 h. However, some literature sources report stability for 24 h at room temperature, even at concentrations higher than those listed in SmPC (Gatti and Pea, 2021a; Loeuille et al., 2022). A study that examined the stability of ceftazidime alone, also confirmed its stability for 24 h at a temperature of up to 25°C within a concentration range 4%–12% (Servais and Tulkens, 2001). Given the discrepancy between the official data in the SmPC and literature sources, and to ensure stability, we decided to conduct a stability study in collaboration with the Faculty of Pharmacy and Biochemistry at the University of Zagreb. The results obtained indicated that solution remained stable for 24 h at room temperature (25°C) when diluted in 0.9% sodium chloride to a final concentration of 40 mg/mL. This confirmed the feasibility of CI over 24 h and led us to the final dosing regimen. Infusion rates, as well as drug doses, are listed in Figure 2. To ensure adherence to infusion protocols, all administrations will be supervised and documented by ICU nursing stuff as part of routine medication charting. Infusion will be delivered using infusion pumps with continuous monitoring capabilities. In addition, a clinical pharmacist, not involved in direct patient care, will verify drug preparation and infusion parameters daily.

Laboratory and microbiological tests will follow the schedule outlined in Table 3. First tests will be run just before the first dose administration, for control and intervention groups. Tests will not be performed if they are available in the previous 24 h. Blood cultures will be performed with two sets. Each set consists of one aerobic and one anaerobic bottle, drawn at the same time from a single venepuncture site. At least one set will not be drawn from the indwelling catheter. Indwelling catheters will be sent for microbiological testing upon removal. Respiratory samples will be taken from lower respiratory tract and will include one of following: sputum, induced sputum, bronchoalveolar lavage (BAL), mini-lavage, endotracheal aspirates (ETA), bronchial washing fluid or pleural fluid, depending on patient’s clinical condition and diagnostic requirements. Urine samples will mostly be taken from urinary catheters, using sterile techniques to minimize contamination and ensure accurate microbiological analysis. Surveillance swabs (nasal, perineal, rectal, axillary) will be taken on a weekly basis.

**TABLE 3:** Laboratory and microbiological tests evaluation schedule.

Day	0	1	2	3	4	5	6	7	8	9	10	11	12	13	14	21	28
Complete blood count	+	+	+	+	+	+	+	+	+	+	+	+	+	+	+	+	+
Arterial blood gas sample^a^	+	+	+	+	+	+	+	+	+	+	+	+	+	+	+		
PT	+	+	+	+	+	+	+	+	+	+	+	+	+	+	+		
aPTT	+	+	+	+	+	+	+	+	+	+	+	+	+	+	+		
Fibrinogen	+	+	+	+	+	+	+	+	+	+	+	+	+	+	+		
Antithrombin activity	+	+	+	+	+	+	+	+	+	+	+	+	+	+	+		
Urinalysis	+							+							+	+	+
Total bilirubin	+	+	+	+	+	+	+	+	+	+	+	+	+	+	+		
Urea	+	+	+	+	+	+	+	+	+	+	+	+	+	+	+	+	+
Creatinine	+	+	+	+	+	+	+	+	+	+	+	+	+	+	+	+	+
eGFR	+	+	+	+	+	+	+	+	+	+	+	+	+	+	+	+	+
AP	+	+	+	+	+	+	+	+	+	+	+	+	+	+	+		
AST	+	+	+	+	+	+	+	+	+	+	+	+	+	+	+		
ALT	+	+	+	+	+	+	+	+	+	+	+	+	+	+	+		
GGT	+	+	+	+	+	+	+	+	+	+	+	+	+	+	+		
K	+	+	+	+	+	+	+	+	+	+	+	+	+	+	+	+	+
Na	+	+	+	+	+	+	+	+	+	+	+	+	+	+	+	+	+
Ca total	+	+	+	+	+	+	+	+	+	+	+	+	+	+	+		
P inorganic	+	+	+	+	+	+	+	+	+	+	+	+	+	+	+		
Mg total	+	+	+	+	+	+	+	+	+	+	+	+	+	+	+		
Proteins	+	+	+	+	+	+	+	+	+	+	+	+	+	+	+		
Albumin	+	+	+	+	+	+	+	+	+	+	+	+	+	+	+		
Pregnancy test^b^	+																
CRP	+	+	+	+	+	+	+	+	+	+	+	+	+	+	+	+	+
PCT	+	+	+	+	+	+	+	+	+	+	+	+	+	+	+	+	+
Blood cultures	+^c^							+^d^							+^d^	+^d^	+^d^
Cultural tests^e^	+							+							+	+	+
Surveillance swabs^f^	+							+							+	+	+
Ceftazidime plasma concentration		+	+	+	+												
MIC^g^	+							+							+	+	+

Abbreviations: PT, prothrombin time; aPTT, activated partial thromboplastin time; eGFR, estimated glomerular filtration rate; AP, alkaline phosphatase; AST, aspartate aminotransferase; ALT, alanine aminotransferase; GGT, gamma-glutamyl transferase; K, potassium, Na, sodium, Ca, calcium; P, phosphorus; Mg, magnesium; CRP, C-reactive protein; PCT, procalcitonin; MIC, minimum inhibitory concentration.

^a^ Arterial blood gas sample and, when available central venous or mixed venous blood gas sample.

^b^ For childbearing age women if there is no previously recorded data.

^c^ If not available in the last 24 h (prior to first drug administration).

^e^ Respiratory and urine samples, indwelling catheters.

^f^ Nasal, perineal, axillary (armpit), rectal.

^g^ MIC of ceftazidime/avibactam for *K. pneumoniae* OXA-48 and *P. aeruginosa*.

^d^ In case of negative blood cultures on study day 0, they will be repeated only if clinically justified.

The duration of treatment will be defined by the clinical judgement of the ICU physician in charge of the patient, in collaboration with a clinical microbiologist. However, some general rules for duration of therapy will be applied.

For patients with microbiologically confirmed diagnosis at the site of infection:

a) Complicated intraabdominal infections: 5–14 days
b) Complicated urinary tract infections: 5–10 days
c) Hospital-acquired pneumonia or ventilator-associated pneumonia: 7–14 days
d) Bacteremia associated with any of the above: in accordance with the primary site of infection
e) G-infections with limited treatment options: guided by the severity of the infection and the patient’s clinical and bacteriological progress (note: there is very limited experience with the use for more than 14 days)

For patients without microbiologically confirmed diagnosis at the site of infection but with at least one other microbiological sample positive for target pathogen:

a) Consider at least 8 days of treatment
b) Consider therapy discontinuation in case of:
  • Clinical resolution of signs and symptoms of infection.
  • Significant improvement in inflammatory biomarkers (e.g., normalization of leukocyte count, CRP and PCT).
  • Negative follow-up microbiological cultures.
  • Resolution of fever, defined as body temperature less than 37.5°C for at least 48 h in the absence of antipyretic therapy.
  • Isolation of an alternative pathogen from the site of infection, not covered by CZA therapy.
  • Lack of further clinical indications to continue therapy, as assessed by the ICU team and microbiologist.

For both groups of patients, therapy adjustments and discontinuation will be guided by daily clinical and microbiological assessments. Prolonged therapy beyond 14 days will require thorough justification, considering factors such as slow clinical progress, unresolved infection or complicating conditions like immunosuppression.

Study will last up to 28 days after the first dose of ceftazidime/avibactam.

#### 2.5.1 Ceftazidime plasma concentration measurement

The concentrations of ceftazidime in human serum will be determined using an in-house high-performance liquid chromatography (HPLC) method with a diode array detector and chloramphenicol as an internal standard. Blood samples will be collected in tubes without anticoagulant and immediately centrifuged at 3,000 rpm for 5 min to separate the serum. Serum sample preparation will be performed by protein precipitation with acetonitrile:methanol (1:1 v/v). The mixture will be briefly vortexed and centrifuged. A phosphate buffer will be added to the supernatant in equal proportions, and after vortexing, the mixture will be injected into the HPLC (Shimadzu, Japan). For the intermittent dosing (ID) group, trough samples will be obtained immediately before the next scheduled dose (i.e., at the end of the 8-h dosing interval, just prior to infusion).

#### 2.5.2 Pathogen identification and antimicrobial susceptibility testing

Samples will be cultured on Columbia blood agar containing 5% sheep blood. Isolate identification will be confirmed by MALDI-TOF mass spectrometry (Bruker Microflex LT, Bremen, Germany). After confirmation of *K. pneumoniae* we will proceed to carbapenemase detection by immunochromatographic test OKNVI resist-5 (Coris BioConcept, Gembloux, Belgium), and confirmation of OXA-48 carbapenemase by PCR testing using OXA-48 primers (Metabion, Planegg, Germany)*.* Confirmed *K. pneumoniae* OXA-48 and *P. aeruginosa* isolates will be submitted to antimicrobial susceptibility testing done by disk diffusion, concentration gradient strip or broth microdilution method and interpreted according to the European Committee on Antimicrobial Susceptibility Testing (EUCAST) criteria. Ceftazidime/avibactam MIC will be determined by commercial concentration gradient strip ETest (bioMérieux, Paris, France) and interpreted according to the EUCAST criteria.

### 2.6 Data collection

The following data will be recorded on study day 0, prior to the first administration of ceftazidime/avibactam. For parameters with multiple available measurements, the values closest to the time of the first dose administration will be used. The schedule for laboratory tests is provided in Table 3.

Data collection:

a) Demographic and administrative data
b) Height and weight
c) Medical history and admission ward
d) Medication history–route and dosage
e) Antimicrobial agents history–route and dosage regimen, including all antimicrobial agents used within 2 months prior to randomization
f) Microbiological isolates history–isolates identified during the current hospitalization
g) Vital signs - systolic and diastolic blood pressure, mean arterial pressure, heart rate, body temperature, central venous pressure
h) Vasoactive-inotropic score
i) SOFA score
j) Glasgow Coma Scale score
k) Ventilatory status - ventilated or not, and whether invasive ventilation is used
l) If ventilated, ventilatory settings
  • Ventilation mode
  • Inspired fraction of oxygen (FiO2)
  • Positive end-expiratory pressure (PEEP)
  • Plateau or maximum inspiratory pressure
  • Mean airway pressure
  • Respiratory rate
  • Dynamic compliance
  • Tidal volume
m) Urine output–over the last 24 h
n) Site of infection - documented or presumed
o) Presence of extracorporeal organ support

The following data will be collected daily from study day 1 through 48 h after the final dose of ceftazidime/avibactam: admission ward, vital signs, medication (route, dosage), vasoactive-inotropic score, SOFA score, new microbiological isolates, ventilatory status, ventilatory settings, urine output, presence of extracorporeal organ support and adverse events.

Microbiological samples will be collected on study days 0, 7, 14, 21 and 28. Samples will also be collected out of schedule (ICU physician or clinical microbiologist discretion) if the patient develops: body temperature above 37.5°C, requirement for introduction of new or higher doses of previously initiated vasopressors/inotropes, worsening of respiratory status, or unexplained worsening in inflammatory biomarkers.

Samples for ceftazidime plasma concentration will be collected on study days 1, 2, 3 and 4. In case of a subsequent need for extracorporeal organ support, samples will also be collected outside the scheduled timeframe.

Total ceftazidime/avibactam dose used will be calculated at the end of administration.

Follow-up on the study day 28 will focus on discharge, survival, and pathogen recurrence rate if not discharged.

All trial data will be recorded in electronic case report forms (eCRFs) using a secure, password-protected platform accessible only to authorized study personnel.

### 2.7 Outcomes

Primary outcome will be microbiological success rate, defined by proportion of patients in whom the causative pathogen is absent from specimen at the site of infection.

Secondary outcomes will be clinical success rate, time to symptoms improvement, length of ICU stay, length of hospital stay, all-cause 28-day mortality after ceftazidime/avibactam initiation, pathogen recurrence rate on day 28, time to weaning from mechanical ventilation, cumulative vasoactive-inotropic score, adverse events, and ratio of ceftazidime plasma concentration to the pathogen’s minimum inhibitory concentration (C/MIC).

### 2.8 Definitions

#### 2.8.1 Study days

Study day 0 is the day of first administration of ceftazidime/avibactam. Study day 1 starts the following day at 6 am, taking into consideration the usual time for blood sampling at the wards.

#### 2.8.2 Intermittent dosing

Intermittent dosing of ceftazidime/avibactam is defined as the standard regimen specified in the SmPC, consisting of 2 g/0.5 g administered every 8 h via an extended infusion over 2 h. The dose is adjusted based on creatinine clearance.

#### 2.8.3 Continuous infusion

Continuous infusion of ceftazidime/avibactam is defined as the delivery of the same total daily dose as would be given with intermittent dosing, administered continuously over 24 h. The preparation of the medication and infusion settings are explained in Figure 2.

#### 2.8.4 Laboratory data

Laboratory parameter values will be reported in the units of measurement used by the local laboratory. Reference ranges for each parameter will also be provided. If necessary for comparison with results from the literature, the values will be converted to ensure consistency. All laboratory tests will be conducted in a single, accredited laboratory, ensuring standardization and consistency of results.

#### 2.8.5 Microbiological data

All microbiological samples positive for *K. pneumoniae* OXA-48 and *P. aeruginosa* will be reported with antimicrobial susceptibility testing interpreted according to EUCAST criteria (European Committee on Antimicrobial Susceptibility Testing, 2024). Ceftazidime/avibactam testing will be reported as MIC and susceptible (S) or resistant (R).

#### 2.8.6 Microbiological success rate

The microbiological success rate is defined as the proportion of patients in whom the causative pathogen is classified as either eradicated or presumed eradicated, as determined by follow-up microbiological assessments and clinical evaluations, evaluated at the end of therapy.

Microbiological outcomes will be categorized as follows:

1. Eradication - The causative pathogen is confirmed to be absent from follow-up microbiological samples.
2. Presumed eradication - Follow-up microbiological samples are not available, but the patient demonstrates sustained clinical improvement without evidence of infection recurrence or relapse, strongly suggesting the pathogen has been eliminated.
3. Persistence - The causative pathogen remains detectable in follow-up microbiological samples despite treatment.
4. Presumed persistence - Follow-up microbiological samples are unavailable, but clinical signs and/or symptoms of infection persist, suggesting the pathogen has not been eradicated.
5. Indeterminate - The microbiological outcome cannot be assessed due to insufficient or missing data, or inconclusive test results.

#### 2.8.7 Clinical success rate

The clinical success rate is defined as the proportion of patients who achieve clinical cure or clinical improvement, evaluated at the end of therapy.

Clinical cure is defined as the complete resolution of all infection-related signs and symptoms, with no evidence of ongoing infection. Clinical improvement is defined as significant reduction in infection-related signs and symptoms such that the patient demonstrates significant progress towards recovery. Failure to achieve clinical success will be classified as clinical failure, which will include any of the following: absence of clinical response (persistent or worsening of infection-related signs or symptoms), therapeutic escalation (need for an unplanned change in antibiotic regimen due to inadequate clinical or microbiological response), infection-related complications (new or worsening organ dysfunction attributable to the infection, dissemination to other body sites, or requirement for surgical or procedural interventions) and microbiological failure (documented persistence or regrowth of the causative organism).

#### 2.8.8 Length of ICU stay

Length of ICU stay is defined as a total number of days from the day of the first CZA administration (study day on 0) until either:

• Discharge from the ICU (if the patient is transferred out of the ICU before day 28)
• Day 28 (for patients who remain in the ICU beyond this time)

It will be calculated with formula:

Length of ICU stay (days) = Date of discharge from ICU or study day 28 − (date of first CZA administration +1).

For patients who die in the ICU, the length of ICU stay will be recorded as the number of days form the first drug administration to date of death.

#### 2.8.9 Length of hospital stay

Length of hospital stay is defined as a total number of days from the day of the first CZA administration (study day on 0) until either:

• Discharge from the hospital (if the patient is discharged before day 28)
• Day 28 (for patients who remain hospitalized beyond this time)

It will be calculated with formula:

Length of hospital stay (days) = Date of discharge or study day 28 − (date of first CZA administration +1).

For patients who die during hospitalization, the length of hospital stay will be recorded as the number of days form the first drug administration to date of death.

#### 2.8.10 All-cause 28-day mortality

All-cause 28-day mortality is defined as the occurrence of death at any point up to and including study day 28. The 28-day mortality rate will be calculated as the number of deaths divided by the total number of patients in the group, expressed as a percentage.

#### 2.8.11 Pathogen recurrence rate on day 28

The pathogen recurrence rate on day 28 is defined as the proportion of patients who have a recurrence of initial causative pathogen from clinical or surveillance microbiological samples taken by study day 28, after completion of CZA therapy.

#### 2.8.12 Vasoactive-inotropic score

Vasoactive-inotropic score is defined as a quantitative measure that evaluates the cumulative effect of vasoactive and inotropic medications. It is calculated using the following formula:

VIS = dobutamine dose (μg/kg/min) + adrenaline dose (μg/kg/min) × 100 + noradrenaline dose (μg/kg/min) × 100 + vasopressin dose (IU/kg/min) × 10 + angiotensin II dose (ng/kg/min) × 10,000.

24 – hour VIS quantifies the overall cardiovascular support required over a 24-h period. Average daily VIS score will be calculated using the following formula:

Daily VIS = ∑ (VIS for each interval × interval duration (hours)/24.

An interval is defined as the period of time where drug doses remain constant.

### 2.9 Sample size estimates

For the planned research, we first performed an *a priori* power calculation using the Z-test for the difference in proportions between two independent groups in G*Power v3.1.9.7 software (Faul et al., 2007). The analysis was performed for a one-sided test with a significance level of 0.05, with expected proportions of *p*_*1*_ = 0.35 for the experimental group, and *p*_*2*_ = 0.65 for the control group. The target power of the test was set at 0.8. The results showed that 34 subjects per group (68 in total) were required to achieve the desired power of 0.8.

To further confirm these results and evaluate the behaviour of the power of the test across different sample sizes, we performed a Monte Carlo simulation in R 4.4.2 (R-Project, 2024). In the simulation, we evaluated the statistical power of a one-sided test of the proportions between two independent groups. The goal of the simulation was to precisely determine the minimum sample size required to achieve a statistical power of at least 0.8 at Cohen’s effect size *h* = 0.3, which corresponds to a medium effect according to Cohen’s classification. The proportions of success in the experimental and control groups were set based on the effect size, where the proportion of success in the experimental group was *p*_*1*_ = 0.5 + h/2, and in the control group *p*_*2*_ = 0.5 − h/2.

For each iteration of the simulation, binary outcomes (success or failure) were generated based on given proportions and sample sizes. We tested samples ranging from 20 to 200 in steps of five. For each sample size, we calculated the difference between the success proportions, corresponding standard error, Z-value, and one-sided p-value. The power of the test was defined as the proportion of iterations in which the p-value was less than a significance level *of α* = 0.05. The simulation involved one million iterations per sample size to ensure stable and accurate estimates.

**FIGURE 3:**
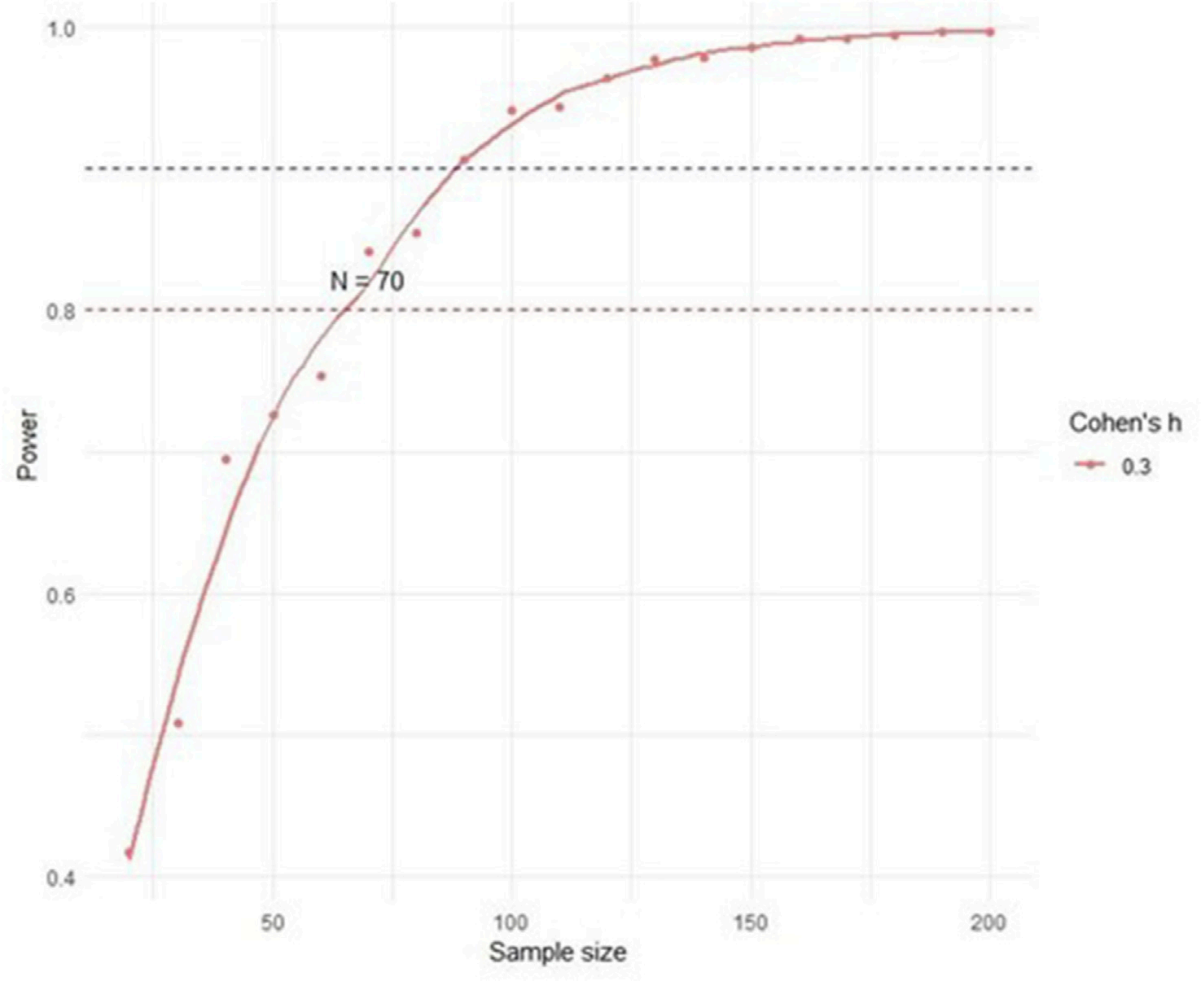
Monte Carlo simulation - power analysis.

The results are graphically presented as a function of the power of the test depending on the sample size, with the power thresholds of 0.8 and 0.9 additionally marked (Figure 3). The resulting minimum sample size for Cohen’s effect size *h* = 0.3 was confirmed as 70 subjects per group.

Finally, we decided to use the sample size obtained by Monte Carlo simulation (70 subjects per group) owing to greater flexibility and precision in power estimation. However, we plan to conduct an interim analysis after including the first 34 subjects in each group to evaluate the preliminary results and decide on the possible need to adjust the sample size.

#### 2.9.1 Statistical plan

The statistical plan includes the application of the Z-test for the difference in proportions for the primary outcome, with a sample size of 70 subjects per group confirmed by Monte Carlo simulation. An interim analysis will be conducted after the inclusion of 34 subjects per group in order to assess the need to adjust the study or stop it earlier. With this approach, we ensure statistical validity, ethical justification and efficient use of resources in the planned research.

Secondary analyses will include a sensitivity assessment by repeating the analysis with different statistical power thresholds, such as 0.9, to test the robustness of the results. We will also calculate confidence intervals for the difference in proportions to better estimate the effect size. If relevant, we will conduct subgroup analyses to consider specific patient characteristics that may influence the outcome.

In addition to the primary outcome, for the secondary outcomes clinical success rate and 28-day mortality, Bonferroni correction for multiple testing will be used to avoid inflation of type I error. The remaining outcomes will be treated as exploratory, with the aim of generating new hypotheses for future research.

Missing microbiological data for the primary endpoint will be conservatively treated as microbiological failure. For secondary outcomes, we will use multiple imputation for variables with missing data >5%, assuming data are missing at random (MAR) and missing completely at random (MCAR). A complete-case sensitivity analysis will be performed to test robustness of the results.

### 2.10 Monitoring of the study

Data entry will be monitored regularly by the clinical trial coordinator and principal investigator for accuracy and completeness. Additionally, the study will be overseen by independent monitors to ensure compliance with national and international regulations, as well as Good Clinical Practice (GCP) guidelines. Monitors will review study processes and data integrity, addressing any critical issues directly with the study team. Data queries will be raised for missing or implausible values and resolved through source data verification. Detailed reports of each monitoring visit will be documented to maintain transparency and accountability throughout the study. Protocol deviations will also be documented, categorized and reviewed monthly by the study team. Major protocol deviations will be reported to the Ethics Committee of UHC Zagreb if they may affect patient safety or study validity. Quality control will be ensured through periodic cross-checking of entries against patient records, and regular internal audits will be performed to detect inconsistencies.

### 2.11 Safety reporting

Considering that ceftazidime/avibactam (Zavicefta) has been authorized since 2016 with a well-established safety profile, and that both study arms will receive the same total daily dose using two clinically accepted dosing regimens, this study will implement a streamlined safety reporting approach in accordance with regulatory and ethical guidelines.

#### 2.11.1 Adverse event (AE) and serious adverse event (SAE) reporting

Serious adverse events (SAEs) will be systematically documented and reported in compliance with ICH E6 (R2) Good Clinical Practice Guidelines. Non-serious AEs will not require routine reporting unless deemed clinically significant or suspected to be directly related to the study intervention. Adverse events that are expected based on the established safety profile of ceftazidime/avibactam, as outlined in the SmPC, will not necessitate expedited reporting unless their nature, frequency, or severity deviates from expectations. Events attributable to underlying infection or pre-existing comorbidities will be assessed separately and will not be classified as study-related unless a direct association with the intervention is established.

#### 2.11.2 Causality assessment and expedited reporting

Investigators will conduct causality assessments for all SAEs to determine their potential relationship to ceftazidime/avibactam administration. Adverse events of special interest (AESIs), including hypersensitivity reactions, neurotoxicity (e.g., encephalopathy, seizures), and hepatic enzyme abnormalities, will be monitored; however, expedited reporting will only be required if their incidence, severity, or presentation is unexpected. SAEs determined to be unrelated to the study drug will be documented but will not be subject to expedited reporting requirements.

#### 2.11.3 Safety monitoring in patients requiring extracorporeal organ upport

In cases where patients require extracorporeal organ support, additional laboratory assessments may be conducted outside the predefined schedule based on clinical necessity.

This risk-adapted safety reporting strategy is designed to ensure regulatory compliance while optimizing the efficiency of data collection, focusing on events that have a direct impact on patient safety and study integrity.

### 2.12 Study location, initiation and timeline

The study will be conducted at the University Hospital Centre Zagreb, Croatia, involving patients from several medical and surgical intensive care units. The limit to the number of units was not set. The study is anticipated to begin in May 2025. The study timeline will be established upon the initiation of the study, with efforts to ensure timely recruitment of eligible patients from the selected ICU settings.

### 2.13 Feasibility

The feasibility of the ZAVICONT trial has been comprehensively assessed to ensure its successful implementation. The target population was identified through a retrospective review, showing an annual average of 36–48 eligible cases of critically ill patients with *K. pneumoniae* OXA-48 or *P. aeruginosa* infections, with an estimated recruitment rate of three to four patients per month, sufficient to achieve the target sample size within 36 months. The trial will be conducted at a tertiary care center with advanced ICU facilities, experienced clinical staff, and an accredited microbiology laboratory capable of rapid diagnostics, as well as accredited Institute for laboratory diagnosis. Stability study conducted in collaboration with the Faculty of Pharmacy and Biochemistry at the University of Zagreb confirmed 24-h stability under trial conditions. The investigators have extensive experience in managing clinical trials, supported by clinical pharmacists and research nurses to ensure adherence to study protocol. Financial resources have been secured through limited institutional support. A pilot simulation confirmed the logistical compatibility of infusion protocols with ICU workflows. The estimated 36-month recruitment period, followed by data analysis and dissemination, aligns with the proposed timeline. Supported by robust institutional resources, experienced personnel, and validated protocols, the ZAVICONT trial is deemed feasible and likely to achieve its objectives.

### 2.14 Significance and expected impact

The ZAVICONT trial addresses a critical gap in the treatment of MDR infections caused by *K. pneumoniae* OXA-48 and *P. aeruginosa* in critically ill patients. Optimizing the administration of CZA through CI versus ID has the potential to enhance drug efficacy by maintaining steady plasma concentrations, improving bacterial eradication, and reducing the emergence of resistance. The findings of this trial could directly shape clinical practice by providing evidence for dosing strategies that maximize therapeutic outcomes. Furthermore, the results may guide updates to treatment guidelines, particularly in intensive care settings, where precision in antimicrobial therapy is essential.

## 3 Discussion

The ZAVICONT trial addresses an urgent need to optimize β-lactam therapy in critically ill patients with severe infections caused by MDR G-bacteria, specifically *K. pneumoniae OXA-48* and carbapenem-resistant *P. aeruginosa*. Due to their limited treatment options, rapid dissemination in hospital settings, and high associated mortality rates, these pathogens pose a significant global threat to public health.

**FIGURE 4:**
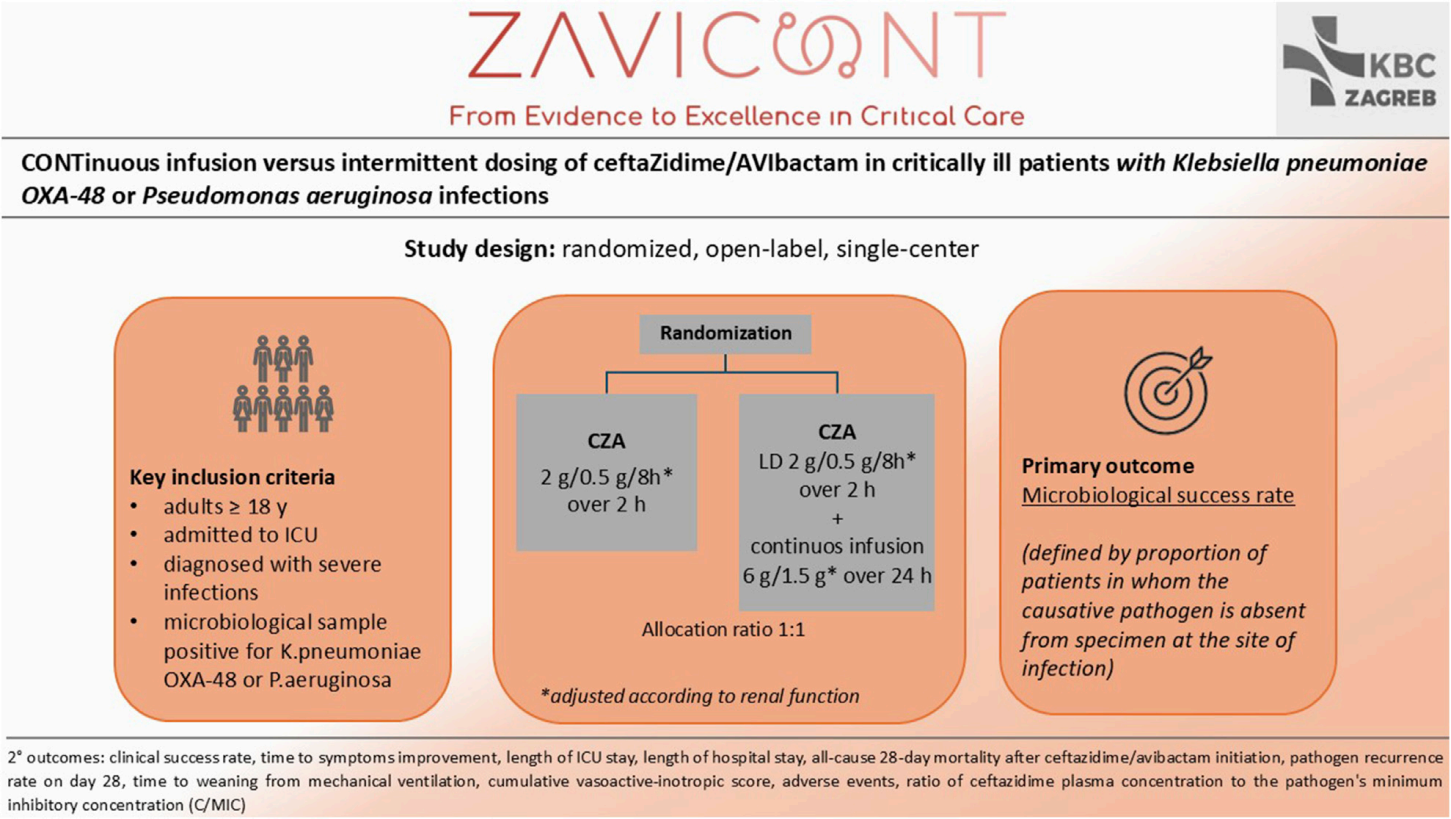
Study design and synopsis of ZAVICONT trial.

Ceftazidime/avibactam has emerged as a crucial treatment for these infections. However, despite its relatively recent clinical introduction, resistance to CZA has already been reported, particularly when suboptimal drug exposure permits pathogens to evolve resistance mechanisms (Cui et al., 2023; Adembri et al., 2020; Gaibani et al., 2021). This is of a special concern in ICU settings, where ID regimens often fail to achieve optimal PK/PD targets due to altered drug PK (Bakdach et al., 2022; Cojutti et al., 2024). The ZAVICONT trial aims to determine whether CI can achieve superior PK/PD outcomes and improve clinical efficacy, leveraging the time-dependent nature of β-lactam activity. This trial builds on previous findings suggesting that CI could be the most effective approach to optimize CZA PK/PD targets, facilitate eradication of DTR G-pathogens, and reduce the risk of microbiological treatment failure while minimizing total daily drug doses (Cojutti et al., 2024; Gatti and Pea, 2021a; Gatti et al., 2023a; Gatti et al., 2023b; Situm et al., 2024). To date, this is one of the first randomized trials specifically evaluating CI versus ID for CZA in critically ill patients (Figure 4), providing much-needed evidence to guide clinical practice in ICU settings.

The primary outcome of the trial, microbiological success, is a crucial endpoint for evaluating pathogen eradication and is directly influenced by achieving adequate PK/PD targets. Secondary outcomes, including clinical success, length of ICU and hospital stay, and all-cause 28-day mortality, will provide a comprehensive assessment of the clinical and economic benefits of CI over ID. The incorporation of the ceftazidime-to-MIC ratio (C/MIC) as a PK/PD marker underscores the trial’s focus on precision medicine, addressing variability in drug exposure among critically ill patients. The trial’s design incorporates practical considerations, such as ensuring the stability of CZA during extended infusions and addressing logistical challenges in ICU environments.

The ZAVICONT trial, while robust, has several limitations that were carefully considered during its design to ensure feasibility and applicability. First, its single-center design may limit the generalizability of findings to other settings with different patient populations, microbiological profiles, or ICU practices. This design choice was made to maintain strict control over patient selection, drug administration, and data collection, ensuring consistency and minimizing variability that could arise from differences in protocols across multiple centers. However, the trial is being conducted in a high-volume tertiary ICU with clinical practices and patient profiles representative of other advanced European ICUs. The study was designed around a clearly defined clinical question, standardized intervention protocols, and commonly encountered pathogens, which enhances its external relevance and provides a solid methodological foundation for future multicenter validation. Second, the lack of blinding could introduce potential bias, particularly in the assessment of subjective outcomes such as clinical success. The decision to not include blinding was based on both logistical and ethical considerations. Administering a placebo alongside the active drug would significantly increase the workload for ICU staff and introduce unnecessary fluid burden in critically ill cardiac patients, many of whom are already at risk of volume overload. To minimize bias, both data collection and outcome assessment will be performed by personnel who are blinded to treatment allocation. Third, the study measures only ceftazidime plasma concentrations, without avibactam quantification. This was due to unavailability of a validated avibactam assay in our country at the time of study initiation. Developing new method would have significantly delayed study initiation. While this approach is consistent with several prior PK studies, we acknowledge that optimal TDM of CZA should ideally assess both components, with reference to publication by Gatti et al. (2024). We plan to implement avibactam monitoring in future studies.

We acknowledge that potential confounders, such as concomitant antimicrobial therapies, variability in ICU practices, and patient-level factors including comorbidities, source control interventions, and organ support could influence study outcomes. To mitigate their impact, eligibility criteria were narrowly defined, and randomization was applied to ensure balance between groups. Moreover, standardized treatment protocols and microbiological procedures were implemented. Relevant concomitant therapies will be documented and considered in sensitivity analyses where applicable. Additionally, we recognize that evolving antimicrobial resistance patterns during the study period may influence microbiological outcomes. This represents an important contextual factor in the interpretation of results, particularly when considering the emergence of new resistance mechanisms to CZA or shifts in local epidemiology.

These design choices ensure that the ZAVICONT trial remains both feasible and highly relevant, with findings that are directly translatable to routine clinical practice while paving the way for future research. Importantly, these limitations do not compromise the study’s internal validity or its ability to address the primary research question, reinforcing the trial’s robustness.

Future directions include prospective multicenter trials to validate these findings across different ICU settings and patient populations. Additional analyses may explore pharmacoeconomic implications, impact on resistance development, and subgroup outcomes based on specific comorbidities or infection sources. By addressing these broader clinical questions, the ZAVICONT trial can serve as a foundation for more personalized and effective β-lactam therapy in critically ill patients.

## 4 Ethics and dissemination

This study will be conducted in accordance with all applicable guidelines for ethical research, including the principles outlined in the Declaration of Helsinki, the International Conference on Harmonisation (ICH) guidelines for Good Clinical Practice (GCP), the Healthcare Act of the Republic of Croatia (NN 100/18), and the Patients' Rights Act of the Republic of Croatia (NN 169/04). The study protocol, participant information sheet, and informed consent form were approved by the Ethics Committee of UHC Zagreb. Prior to enrollment, all participants will provide written informed consent. Given the critically ill nature of the participant population, the use of deferred consent will be applied when the participant is temporarily unable to provide consent, in accordance with national regulations and Article 30 of the Declaration of Helsinki. In such cases, informed consent will be obtained from a legally authorized representative. If no representative is immediately available and if inclusion in the study cannot be delayed, the patient may be enrolled without prior consent, provided that this is explicitly justified in the protocol and the procedure has received prior Ethics Committee approval. Consent to remain in the study will be sought as soon as possible from the patient or their legal representative. Additional safeguards will be implemented to ensure that participants and/or their representatives fully understand the potential risks and benefits of participation. Participant data confidentiality will be strictly maintained by assigning each participant a unique numerical code for data storage in electronic databases. Access to sensitive data will be restricted to authorized personnel only. A risk-adapted safety monitoring strategy has been introduced and is detailed in the Methods section.

The findings of the ZAVICONT trial will be shared widely to maximize their impact on clinical practice and scientific knowledge. Results will be published in peer-reviewed journals focused on infectious diseases, critical care, and pharmacology to ensure broad audience within relevant fields. Additionally, the study team will present key results at both national and international conferences.

## Glossary

% fT: percent time of the free-drug concentration
AE: adverse event
AESI: adverse event of special interest
ALT: alanine aminotransferase
AP: alkaline phosphatase
aPTT: activated partial thromboplastin time
AST: aspartate aminotransferase
BAL: bronchoalveolar lavage
BSIs: bloodstream infections
Ca: calcium
CI: continuous infusion
cIAI: complicated intraabdominal infections
Cl: clearance
CrCl: creatinine clearance
CRKP: carbapenem resistant *Klebisella Pneumoniae*
CRP: C-reactive protein
CRRT: continuous renal replacement therapy
Ct: concentration threshold
cUTI: complicated urinary tract infections
CZA: CeftaZidime/Avibactam
DTR: difficult-to-treat
ECDC: European Centre for Disease Prevention and Control
eCRF: electronic Case Report Form
eGFR: estimated Glomerular Filtration Rate
EMA: European Medicines Agency
ETA: EndoTracheal Aspirates
EARS-Net: European Antimicrobial Resistance Surveillance Network
FDA: Food and Drug Administration
G (−): Gram-Negative
GCP: good clinical practice
GGT: gamma-glutamyl transferase
HAP: Hospital Aquired Pneumonia
HPLC: high performance liquid chromatography
ICH: International Conference on Harmonisation
ICU: intensive care unit
K: potassium
KP: *Klebsiella pneumoniae*
KPC: *Klebsiella Pneumoniae* Carbapenemase
MBL: metallo-β-lactamase
MAR: missing at random
MCAR: missing completely at random
MDR: multidrug-resistant
Mg: magnesium
MIC: minimum inhibitory concentration
Na: sodium
NDM: New Delhi Metallo-β-lactamase
OXA-48: OXAcillinase-48
P: phosphorus
PCT: ProCalciTonine
PICO: population, intervention, comparator, outcome
PK/PD: PharmacoKinetic/PharmacoDynamic
PT: prothrombin time
SAE: severe adverse event
SmPC: summary of product characteristics
ST: sequence type
t1/2: half-life time
TDM: therapeutic drug monitoring
UHC: University Hospital Centre
VAP: ventilator associated pneumonia
Vd: volume of distribution
VIS: vasoactive-inotropic score
WHO: World Health Organisation

## References

[B1] Adembri C. Novelli A. Nobili S. (2020). Some suggestions from PK/PD principles to contain resistance in the clinical setting – focus on ICU patients and Gram-negative strains. Antibiotics 9 (10), 676. 10.3390/antibiotics9100676 33036190 PMC7601871

[B2] Ali A. Mudassar I. Sial S. Khan A. (2022). Effective antibiotic dosing in the presence of resistant strains. PLoS One 17 (10), e0275762. 10.1371/journal.pone.0275762 36215219 PMC9551627

[B3] Al-Shaer M. H. Rubido E. Cherabuddi K. Venugopalan V. Klinker K. Peloquin C. (2020). Early therapeutic monitoring of β-lactams and associated therapy outcomes in critically ill patients. J. Antimicrob. Chemother. 75 (12), 3644–3651. 10.1093/jac/dkaa359 32910809

[B4] Angus B. J. Smith M. D. Suputtamongkol Y. Mattie H. Walsh A. L. Wuthiekanun V. (2000). Pharmacokinetic-pharmacodynamic evaluation of ceftazidime continuous infusion vs intermittent bolus injection in septicaemic melioidosis. Br. J. Clin. Pharmacol. 50, 184–191. 10.1111/j.1365-2125.2000.00179.x 10930972 PMC2014399

[B5] Bakdach D. Elajez R. Bakdach A. R. Awaisu A. De Pascale G. Hssain A. A. (2022). Pharmacokinetics, pharmacodynamics, and dosing considerations of novel β-lactams and β-lactam/β-lactamase inhibitors in critically ill adult patients: focus on obesity, augmented renal clearance, renal replacement therapies, and extracorporeal membrane oxygenation. J. Clin. Med. 11 (23), 6898. 10.3390/jcm11236898 36498473 PMC9738279

[B6] Batchelder J. I. Hare P. J. Mok W. W. K. (2023). Resistance-resistant antibacterial treatment strategies. Front. Antibiot. 2, 1093156. 10.3389/frabi.2023.1093156 36845830 PMC9954795

[B7] Berkhout J. Melchers M. J. van Mil A. C. Seyedmousavi S. Lagarde C. M. Schuck V. (2016). Pharmacodynamics of ceftazidime and avibactam in neutropenic mice with thigh or lung infection. Antimicrob. Agents Chemother. 60, 368–375. 10.1128/AAC.01269-15 26525790 PMC4704241

[B8] Bonomo R. A. Burd E. M. Conly J. Limbago B. Poirel L. Segre J. A. (2018). Carbapenemase-producing organisms: a global scourge. Clin. Infect. Dis. 66 (8), 1290–1297. 10.1093/cid/cix893 29165604 PMC5884739

[B9] Carmeli Y. Armstrong J. Laud P. J. Newell P. Stone G. G. Wardman A. (2016). Ceftazidime-avibactam or best available therapy in patients with ceftazidime-resistant *Enterobacteriaceae* and *Pseudomonas aeruginosa* complicated urinary tract infections or complicated intra-abdominal infections (REPRISE): a randomized, pathogen-directed, phase 3 study. Lancet Infect. Dis. 16, 661–673. 10.1016/S1473-3099(16)30004-4 27107460

[B10] Ceron S. Salem-Bango Z. Contreras D. A. Ranson E. L. Yang S. (2023). Clinical and genomic characterisation of carbapenem-resistant *Klebsiella pneumoniae* with concurrent production of NDM and OXA-like-carbapenemases in Southern California, 2016–2022. Microorganisms 11 (7), 1717. 10.3390/microorganisms11071717 37512889 PMC10383945

[B11] Chen T. Xu H. Chen Y. Ji J. Ying C. Liu Z. (2023). Identification and characterization of OXA-232-producing sequence type 231 multidrug resistant *Klebsiella pneumoniae* strains causing bloodstream infections in China. Microbiol. Spectr. 11 (2), e0260722. 10.1128/spectrum.02607-22 36946763 PMC10100818

[B12] Cojutti P. G. Pai M. P. Gatti M. Rinaldi M. Ambretti S. Viale P. (2024). An innovative population pharmacokinetic/pharmacodynamic strategy for attaining aggressive joint PK/PD target of continuous infusion ceftazidime/avibactam against KPC- and OXA-48-producing Enterobacterales and preventing resistance development in critically ill patients. J. Antimicrob. Chemother*.* 79, 2801–2808. 10.1093/jac/dkae290 39159014

[B13] Coleman K. Levasseur P. Girard A. M. Borgonovi M. Miossec C. Merdjan H. (2014). Activities of ceftazidime and avibactam against beta-lactamase-producing *Enterobacteriaceae* in a hollow-fiber pharmacodynamic model. Antimicrob. Agents Chemother. 58, 3366–3372. 10.1128/AAC.00080-14 24687507 PMC4068505

[B14] Cousson J. Floch T. Vernet-Garnier V. Apprioru M. Petit J. S. Jovenin N. (2005). Pharmacodynamic interest of ceftazidime continuous infusion vs intermittent bolus administration in patients with severe nosocomial pneumonia. Pathol. Biol. 53, 546–550. 10.1016/j.patbio.2005.06.002 16023303

[B15] Cui Q. Wang C. Wang Q. Qin J. Li M. Ding B. (2023). Ceftazidime/avibactam resistance in carbapenemase-producing *Klebsiella pneumoniae* . Emerg. Infect. Dis. 29 (11), 2398–2400. 10.3201/eid2911.230830 37877674 PMC10617341

[B16] Das S. Li J. Riccobene T. Carrothers T. J. Newell P. Melnick D. (2019). Dose selection and validation for ceftazidime-avibactam in adults with complicated intra-abdominal infections, complicated urinary tract infections, and nosocomial pneumonia. Antimicrob. Agents Chemother. 63 (4), e02187-18. 10.1128/AAC.02187-18 30670413 PMC6437548

[B17] De Jonge B. L. M. Karlowsky J. A. Kazmierczak K. M. Biedenbach D. J. Sahm D. F. Nichols W. W. (2016). In vitro susceptibility to ceftazidime-avibactam of carbapenem-nonsusceptible *Enterobacteriaceae* isolates collected during the INFORM global surveillance study (2012 to 2014). Antimicrob. Agents Chemother. 60, 3163–3169. 10.1128/AAC.03042-15 26926648 PMC4862516

[B18] El Haj C. Benavent E. Soldevila-Boixader L. Rigo-Bonnin R. Tubau F. Torrejon B. (2024). Comparative efficacy of continuous ceftazidime infusion vs. intermittent bolus against in vitro ceftazidime-susceptible and -Resistant *Pseudomonas aeruginosa* Biofilm. Antibiotics 13, 344. 10.3390/antibiotics13040344 38667020 PMC11047404

[B19] European Centre for Disease Prevention and Control (ECDC) (2023a). Antimicrobial resistance in the EU/EEA (EARS-Net) - annual epidemiological report for 2023. Available online at: https://www.ecdc.europa.eu/en/publications-data/antimicrobial-resistance-eueea-ears-net-annual-epidemiological-report-2023 (Accessed July 6, 2025).

[B20] European Centre for Disease Prevention and Control (ECDC) (2023b). Country summaries - antimicrobial resistance in the EU/EEA 2023. Available online at: https://www.ecdc.europa.eu/sites/default/files/documents/Country_profiles_2023_2024_1.pdf (Accessed July 6, 2025).

[B21] European Committee on Antimicrobial Susceptibility Testing (2024). Breakpoint tables for interpretation of MICs and zone diameters version 14.0. Available online at: https://www.eucast.org/fileadmin/src/media/PDFs/EUCAST_files/Breakpoint_tables/v_14.0_Breakpoint_Tables.pdf (Accessed November 13, 2024).

[B22] European Medicines Agency (2024). Summary of product characteristics – zavicefta 2 g/0.5 g powder for concentrate for solution for infusion. Available online at: https://www.ema.europa.eu/en/documents/product-information/zavicefta-epar-product-information_en.pdf (Accessed November 11, 2024).

[B23] Falcone M. Paterson D. (2016). Spotlight on ceftazidime/avibactam: a new option for MDR Gram-negative infections. J. Antimicrob. Chemother. 71, 2713–2722. 10.1093/jac/dkw239 27432599

[B24] Faul F. Erdfelder E. Lang A.-G. Buchner A. (2007). G*Power 3: a flexible statistical power analysis program for the social, behavioral, and biomedical sciences. Behav. Res. Methods 39 (2), 175–191. 10.3758/BF03193146 17695343

[B25] Flamm R. K. Stone G. G. Sader H. S. Jones R. N. Nichols W. W. (2014). Avibactam reverts the ceftazidime MIC90 of European Gram-negative bacterial clinical isolates to the epidemiological cutoff value. J. Chemother. 26, 333–338. 10.1179/1973947813Y.0000000145 24125508

[B26] Fresan D. Luque S. Benitez-Cano A. Sorli L. Montero M. M. De-Antonio M. (2023). Pharmacokinetics/pharmacodynamics and therapeutic drug monitoring of ceftazidime/avibactam administered by continuous infusion in patients with MDR Gram-negative bacterial infections. J. Antimicrob. Chemother. 78, 678–683. 10.1093/jac/dkac439 36626402

[B27] Gaibani P. Gatti M. Rinaldi M. Crovara Pesce C. Lazzaroto T. Giannella M. (2021). Suboptimal drug exposure leads to selection of different subpopulations of ceftazidime-avibactam-resistant *Klebsiella pneumoniae* carbapenemase-producing *Klebsiella pneumoniae* in a critically ill patient. Int. J. Infect. Dis. 113, 213–217. 10.1016/j.ijid.2021.10.028 34656787

[B28] Garcia-Gonzalez N. Fuster B. Tormo N. Salvador C. Gimeno C. Gonzalez-Candelas F. (2023). Genomic analysis of the initial dissemination of carbapenem-resistant *Klebsiella pneumoniae* clones in a tertiary hospital. Microb. Genom. 9 (6), 001032. 10.1099/mgen.0.001032 PMC10327503 37272914

[B29] Gatti M. Pascale R. Cojutti P. G. Rinaldi M. Ambretti S. Conti M. (2023a). A descriptive pharmacokinetic/pharmacodynamic analysis of continuous infusion ceftazidime-avibactam in a case series of critically ill renal patients treated for documented carbapenem-resistant Gram-negative bloodstream infections and/or ventilator-associated pneumonia. Int. J. Antimicrob. Agents 61 (1), 106699. 10.1016/j.ijantimicag.2022.106699 36464151

[B30] Gatti M. Pea F. (2021a). Pharmacokinetic/pharmacodynamic target attainment in critically ill renal patients on antimicrobial usage: focus on novel beta-lactams and beta-lactams/beta-lactamase inhibitors. Expert Rev. Clin. Pharmacol. 14 (5), 583–599. 10.1080/17512433.2021.1901574 33687300

[B31] Gatti M. Pea F. (2021b). Continuous versus intermittent infusion of antibiotics in Gram-negative multidrug-resistant infections. Curr. Opin. Infect. Dis. 34 (6), 737–747. 10.1097/QCO.0000000000000755 34261906

[B32] Gatti M. Rinaldi M. Gaibani P. Siniscalchi A. Tonetti T. Giannella M. (2023b). A descriptive pharmacokinetic/pharmacodynamic analysis of continuous infusion ceftazidime-avibactam for treating DTR gram-negative infections in a case series of critically ill patients undergoing continuous veno-venous haemodiafiltration (CVVHDF). J. Crit. Care 76, 154301. 10.1016/j.jcrc.2023.154301 37059003

[B33] Gatti M. Viale P. Pea F. (2024). Therapeutic drug monitoring of ceftazidime/avibactam: why one leg is not enough to run. J. Antimicrob. Chemother. 79, 195–199. 10.1093/jac/dkad367 38019676

[B34] Ghazi I. M. El Nekidy W. S. (2023). Editorial: advances in antimicrobial therapy and combating resistance. Front. Pharmacol. 14, 1170289. 10.3389/fphar.2023.1170289 37007043 PMC10061092

[B35] Gómez C. M. Cordingly J. J. Palazzo M. G. (1999). Altered pharmacokinetics of ceftazidime in critically ill patients. Antimicrob. Agents Chemother. 43, 1798–1802. 10.1128/AAC.43.7.1798 10390248 PMC89369

[B36] Kazmierczak K. M. de Jonge B. L. M. Stone G. G. Sahm D. F. (2018). In vitro activity of ceftazidime/avibactam against isolates of *Enterobacteriaceae* collected in European countries: INFORM global surveillance 2012–15. J. Antimicrob. Chemother. 73 (10), 2782–2788. 10.1093/jac/dky266 30010894

[B37] Li M. Gao L. Wang Z. Zeng L. Chen C. Wang J. (2024). Population pharmacokinetics and dose optimization of ceftazidime in critically ill children. Front. Pharmacol. 14, 1470350. 10.3389/fphar.2024.1470350 PMC11631598 39664522

[B38] Loeuille G. D’Huart E. Vingeron J. Nisse Y.-E. Beiler B. Polo C. (2022). Stability studies of 16 antibiotics for continuous infusion in intensive care units and for performing outpatient parenteral antimicrobial therapy. Antibiotics 11, 458. 10.3390/antibiotics11040458 35453211 PMC9030478

[B39] Lorente L. Jimenez A. Palermo S. Jimenez J. J. Iribarren J. L. Santana M. (2007). Comparison of clinical cure rates in adults with ventilator-associated pneumonia treated with intravenous ceftazidime administered by continuous or intermittent infusion: a retrospective, nonrandomized, open-label, historical chart review. Clin. Ther. 29 (11), 2433–2439. 10.1016/j.clinthera.2007.11.003 18158083

[B40] Mazuski J. E. Gasink L. B. Armstrong J. Broadhurst H. Stone G. G. Rank D. (2016). Efficacy and safety of ceftazidime-avibactam plus metronidazole versus meropenem in the treatment of complicated intra-abdominal infection: results from a randomized, controlled, double-blind, phase 3 program. Clin. Infect. Dis. 62 (11), 1380–1389. 10.1093/cid/ciw133 26962078 PMC4872289

[B41] McNabb J. J. Nightingale C. H. Quintiliani R. Nicolau D. P. (2001). Cost-effectiveness of ceftazidime by continuous infusion versus intermittent infusion for nosocomial pneumonia. Pharmacotherapy 21, 549–555. 10.1592/phco.21.6.549.34539 11349744

[B42] Muller A. E. Punt N. Mouton J. W. (2013). Optimal exposures of ceftazidime predict the probability of microbiological and clinical outcome in the treatment of nosocomial pneumonia. J. Antimicrob. Chemother. 68, 900–906. 10.1093/jac/dks468 23190766

[B43] Nichols W. W. de Jonge B. L. Kazmierczak K. M. Karlowsky J. A. Sahm D. F. (2016). In vitro susceptibility of global surveillance isolates of *Pseudomonas aeruginosa* to ceftazidime-avibactam: INFORM 2012–2014. Antimicrob. Agents Chemother. 22, 4743–4749. 10.1128/AAC.00220-16 PMC4958170 27216074

[B44] Nicolau D. P. McNabb J. Lacy M. K. Quintiliani R. Nightingale C. H. (2001). Continuous versus intermittent administration of ceftazidime in intensive care unit patients with nosocomial pneumonia. Int. J. Antimicrob. Agents 17 (6), 497–504. 10.1016/s0924-8579(01)00329-6 11397621

[B45] Poole K. (2011). *Pseudomonas aeruginosa*: resistance to the max. Front. Microbiol. 2, 65. 10.3389/fmicb.2011.00065 21747788 PMC3128976

[B46] Qin X. Tran B. G. Kim M. J. Kim H. Wang H. Nguyen D. A. (2017). A randomised, double-blind, phase 3 study comparing the efficacy and safety of ceftazidime/avibactam plus metronidazole versus meropenem for complicated intra-abdominal infections in hospitalised adults in Asia. Int. J. Antimicrob. Agents 49, 579–588. 10.1016/j.ijantimicag.2017.01.010 28363526

[B47] Roberts J. A. Paul S. K. Akova M. Bassetti M. de Waele J. J. Dimopoulos G. (2014). DALI: defining antibiotic levels in intensive care unit patients: are current β-lactam antibiotic doses sufficient for critically ill patients? Clin. Infect. Dis. 58 (8), 1072–1083. 10.1093/cid/ciu027 24429437

[B48] Rondanelli R. Dionigi R. V. Regazzi M. B. Maurelli M. Calvi M. Mapelli A. (1986). Ceftazidime in the treatment of *Pseudomonas* infections in intensive-care patients. Int. J. Clin. Pharmacol. Ther. Toxicol. 24, 457–459. 3536763

[B49] R Project (2024). The R project for statistical computing. Available online at: https://www.r-project.org/ (Acessed November 25, 2024).

[B50] Servais H. Tulkens P. M. (2001). Stability and compatibility of ceftazidime administered by continuous infusion to intensive care patients. Antimicrob. Agents Chemother. 45, 2643–2647. 10.1128/AAC.45.9.2643-2647.2001 11502544 PMC90707

[B51] Situm I. Karmelic D. Mandaric A. Erceg A. Lovric D. Siroglavic M. (2024). Effectiveness of ceftazidime/avibactam as continuous infusion in critically ill patients with OXA-48-producing *Klebsiella pneumoniae* infection. Ro. Med. J. 71 (2), 156–159. 10.37897/RMJ.2024.2.15

[B52] Tamma P. D. Bergman Y. Jacobs E. B. Lee J. H. Lewis S. Cosgrove S. E. (2023). Comparing the activity of novel antibiotic agents against carbapenem resistant *Enterobacteriaceae* clinical isolates. Infect. Control. Hosp. Epidemiol. 44 (5), 762–767. 10.1017/ice.2022.161 35822340

[B53] Torres A. Wible M. Tawadrous M. Irani P. Stone G. G. Quintana A. (2023). Efficacy and safety of ceftazidime/avibactam in patients with infections caused by β-lactamase-producing Gram-negative pathogens: a pooled analysis from the phase 3 clinical trial programme. J. Antimicrob. Chemother. 78, 2672–2682. 10.1093/jac/dkad280 37700689 PMC11157139

[B54] Torres A. Zhong N. Pachl J. Timsit J.-F. Kollef M. Chen Z. (2018). Ceftazidime-avibactam versus meropenem in nosocomial pneumonia, including ventilator-associated pneumonia (REPROVE): a randomised, double-blind, phase 3 non-inferiority trial. Lancet Infect. Dis. 18, 285–295. 10.1016/S1473-3099(17)30747-8 29254862

[B55] Wagenlehner F. M. Sobel J. D. Newell P. Armstrong J. Huang X. Stone G. G. (2016). Ceftazidime-avibactam versus doripenem for the treatment of complicated urinary tract infections, including acute pyelonephritis: recapture, a phase 3 randomized trial program. Clin. Infect. Dis. 63, 754–762. 10.1093/cid/ciw378 27313268 PMC4996135

[B56] World Health Organization (WHO) (2024). WHO bacterial priority pathogens list, 2024: bacterial pathogens of public health importance to guide research, development and strategies to prevent and control antimicrobial resistance. Available online at: https://www.who.int/publications/i/item/9789240093461 (Accessed November 11, 2024).

[B57] Young R. J. Lipman J. Gin T. Gomersall C. D. Joynt G. M. Oh T. E. (1997). Intermittent bolus dosing of ceftazidime in critically ill patients. J. Antimicrob. Chemother. 40, 269–273. 10.1093/jac/40.2.269 9301994

